# Case report: Pathological complete response to neoadjuvant brigatinib in stage III non-small cell lung cancer with *ALK* rearrangement

**DOI:** 10.3389/fonc.2024.1343238

**Published:** 2024-07-11

**Authors:** Hayoung Seong, Soo Han Kim, Mi Hyun Kim, Jeong Su Cho, Ahrong Kim, Jung Seop Eom

**Affiliations:** ^1^ Department of Internal Medicine, Pusan National University School of Medicine, Busan, Republic of Korea; ^2^ Department of Internal Medicine, Pusan National University Hospital, Busan, Republic of Korea; ^3^ Department of Thoracic and Cardiovascular Surgery, Pusan National University Hospital, Busan, Republic of Korea; ^4^ Department of Pathology, Pusan National University Hospital, Busan, Republic of Korea; ^5^ Biomedical Research Institute, Pusan National University Hospital, Busan, Republic of Korea

**Keywords:** lung cancer, anaplastic lymphoma kinase-tyrosine kinase inhibitor, brigatinib, neoadjuvant treatment, case report

## Abstract

**Purpose:**

The use of neoadjuvant anaplastic lymphoma kinase (ALK)-tyrosine kinase inhibitors (TKIs) has not been extensively explored. The current case report highlights the notable pathological complete response (pCR) achieved following neoadjuvant brigatinib therapy in a patient with stage IIIA ALK-positive non-small cell lung cancer (NSCLC).

**Case presentation:**

A 32-year-old male presented with incidental lung lesions, ultimately diagnosed as clinical stage T3N1M0, IIIA NSCLC with an *ALK* gene rearrangement. Following a multidisciplinary discussion, the patient opted for neoadjuvant brigatinib therapy, which significantly reduced the tumor size. Subsequently, surgery with curative intent was performed, revealing pCR with no residual tumor cells. The patient remained disease-free during a 13-month follow-up period.

**Conclusion:**

This case report provides compelling evidence of pCR following brigatinib therapy in ALK-positive NSCLC, suggesting that surgery after neoadjuvant therapy with brigatinib may offer a safe and effective approach for patients with ALK-positive NSCLC.

## 1 Introduction

Advances in targeted therapy and immunotherapy have led to personalized treatment approaches for advanced non-small cell lung cancer (NSCLC) (1, 2). In particular, the introduction of anaplastic lymphoma kinase (ALK)-tyrosine kinase inhibitors (TKIs) has transformed the treatment possibilities for patients with advanced NSCLC, with *ALK* gene rearrangements, affording an objective response rate of 74% and prolonged survival (3, 4).

Evidence from recent trials suggests the potential benefits of neoadjuvant or adjuvant targeted therapy in early-stage disease (5, 6). However, the role of ALK-TKIs in early-stage and locally advanced ALK-positive NSCLC has not been extensively analyzed. Certain retrospective studies and small-scale, early-phase trials have reported excellent responses to the neoadjuvant use of ALK-TKIs (7–9).

Brigatinib, a second-generation ALK-TKI, has shown remarkable efficacy in treating advanced ALK-positive NSCLC by overcoming crizotinib-resistant *ALK* mutations (10). Herein, we present, to the best of our knowledge, the first case report of a patient achieving a pathologic complete response (pCR) after brigatinib therapy in the neoadjuvant setting of stage III ALK-positive NSCLC. The present case not only highlights the safety and feasibility of surgery following brigatinib therapy but also affords pathological evidence of the potent antitumor efficacy of brigatinib.

## 2 Case description

A 32-year-old male with no comorbidities, a history of only two pack-years of current smoking, and without special risk factors, such as a family history of lung cancer, occupational exposure, or environmental exposure, visited a tertiary referral hospital for further evaluation of incidental lung nodules. The nodules had been discovered on a chest X-ray during a routine health check-up in November 2022, and he did not exhibit any specific symptoms. Serum tumor marker analysis revealed an elevated carcinoembryonic antigen (CEA) level of 8.49 ng/ml (reference value < 5 ng/ml). However, routine blood chemistry and pulmonary function tests showed no significant abnormalities.

## 3 Diagnostic assessment

**Figure 1:**
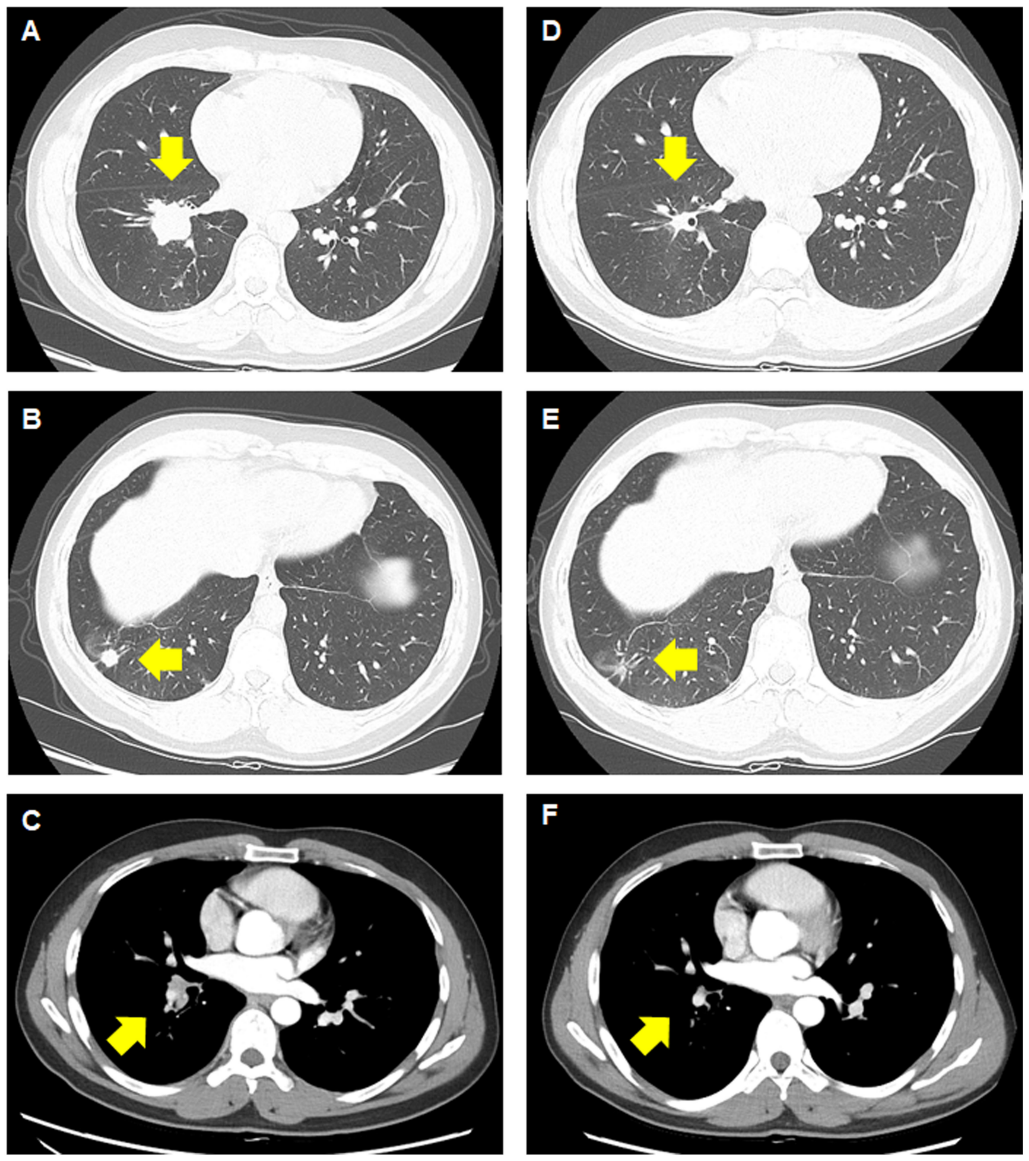
Computed tomography images before and after treatment with brigatinib. **(A–C)** Before treatment, a primary mass measuring 4.9 cm can be observed, along with a satellite nodule measuring 1.2 cm in the lower lobe of the right lung, with right interlobar lymph node metastasis. **(D–F)** A significant response in the primary lung lesions, satellite nodule, and lymph node can be observed after brigatinib therapy.

**Figure 2:**
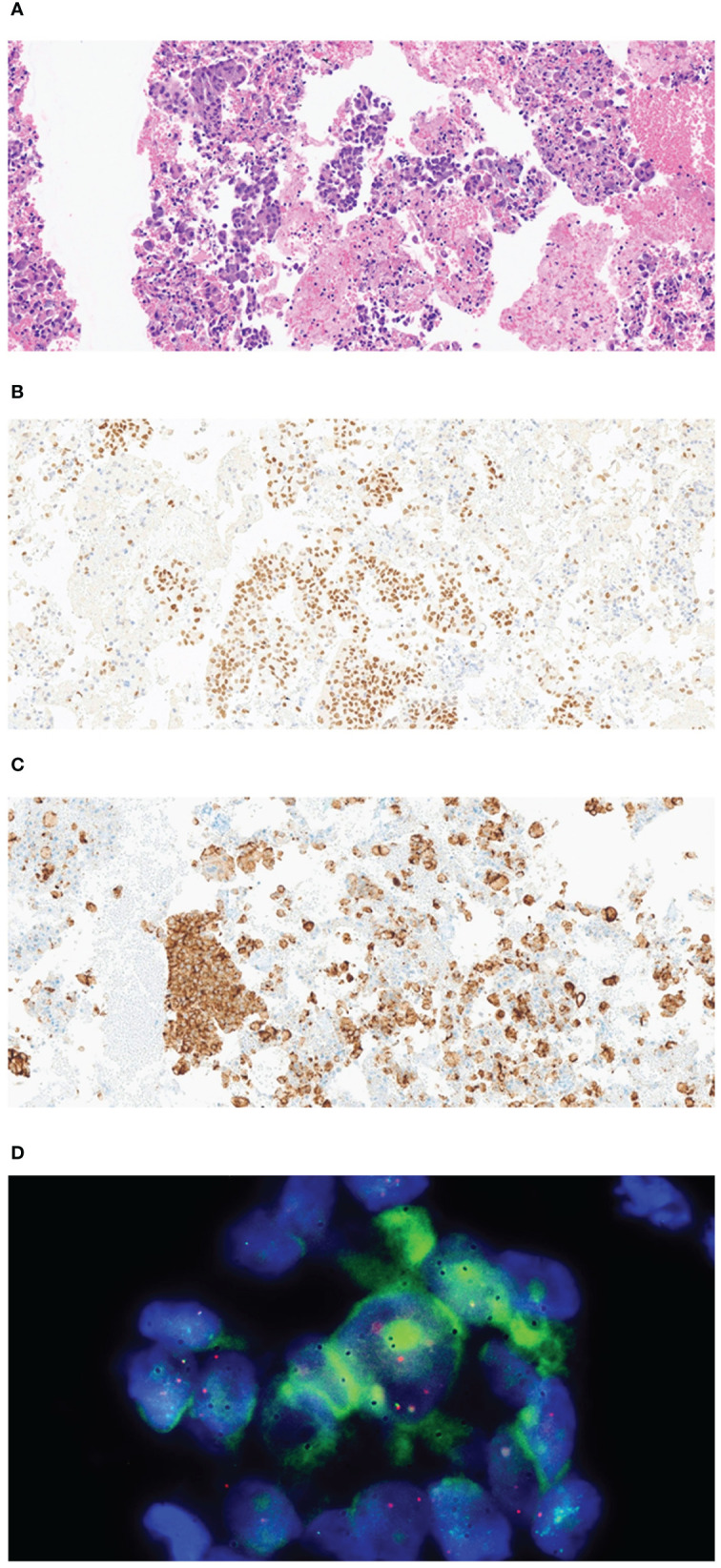
Pathological examination of the tissue specimen from endobronchial ultrasound-guided transbronchial needle aspiration and video-assisted thoracoscopic right lower lobectomy with mediastinal lymph node dissection. **(A)** The biopsy sample shows clusters of atypical epithelial cells with plump cytoplasm (H&E, 200×). **(B)** Representative immunohistochemistry image showing tumor cells nuclear-positive for TTF1 (200×). **(C)** Representative image showing strong granular cytoplasmic expression of anaplastic lymphoma kinase (D5F3, Ventana CDx) (H&E, 200×). **(D)** Representative image showing a break in the ALK locus, indicated by the Vysis ALK Break abnormal signal pattern of isolated red and distinct break-apart signals (Vysis ALK Break Apart FISH probe).

**Figure 3:**
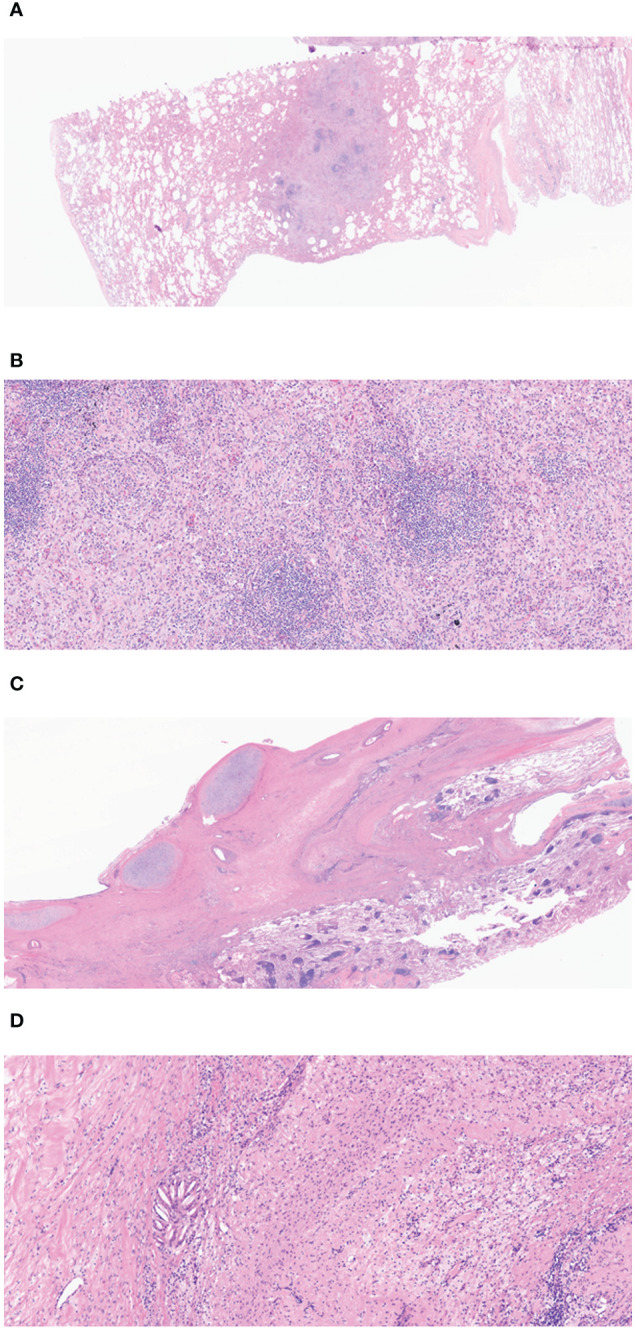
**(A)** The low magnification view shows a nodular area, considered the tumor bed (H&E, 10×). **(B)** Lymphoid aggregation and fibroblast can be observed in the nodular area of **(A)** (H&E, 100×). **(C)** Fibrotic changes can be seen in the peribronchial area, which is compatible with the tumor bed (H&E, 400×). **(D)** In the high-power view of **(C)**, macrophage and lymphocyte infiltration and a cholesterol cleft can be observed in the fibrotic area. (H&E). Overall, there are no viable tumor cells on tumor bed and the final diagnosis is compatible with pathologic complete response.

**Figure 4:**
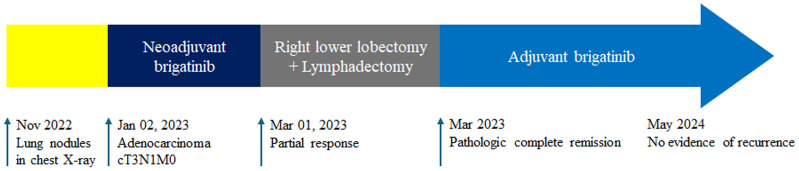
The timeline of the entire therapy process.

On chest computed tomography, two lobulated enhancing lung lesions, measuring 4.9 and 1.2 cm, were identified in the right lower lobe, along with an enlarged right interlobar lymph node (**Figures 1A–C**). No other significant findings were noted on the computed tomography. Endobronchial ultrasound-guided transbronchial needle aspiration was performed on the right lower lobe mass. Histopathological examination of the biopsy specimen revealed adenocarcinoma with focal mucinous differentiation (**Figures 2A, B**). During staging investigations, no evidence of distant metastasis was observed on the bone scan, brain magnetic resonance imaging, and positron emission tomography. Ultimately, the patient was diagnosed with clinical stage T3N1M0, IIIA (American Joint Committee on Cancer 8^th^ edition) on January 2, 2023 (11). Molecular testing using both immunohistochemistry, with a monoclonal antibody (D5F3; Ventana-Roche Diagnostics, Mannheim, Germany), and fluorescent *in situ* hybridization (Vysis LSI ALK Break-Apart FISH Probe Kit, Abbott Molecular Inc., Abbott Park, IL, USA) showed an *ALK* gene rearrangement (**Figures 2C, D**). After a multidisciplinary discussion, the patient decided to proceed with surgery, after neoadjuvant brigatinib therapy. Brigatinib monotherapy was administered at a dose of 90 mg, once daily for the first 7 days. This was well-tolerated; the dose was subsequently increased to 180 mg once daily and continued. This treatment provided a dramatic radiographic response, with a significant reduction in the primary lung tumor size from 4.9 to 1.7 cm (**Figures 1D–F**). Upon re-evaluation of serum tumor markers, CEA was reduced to below-normal levels, and there were no abnormalities detected in other routine blood biochemistry tests. After 58 days of brigatinib therapy, video-assisted thoracoscopic right lower lobectomy with mediastinal lymph node dissection was performed with curative intent. The surgical procedure was performed without complications, with no residual tumor detected on analysis of intraoperative frozen sections. Histopathological examination of the resected specimen then confirmed a pCR, with no evidence of viable tumor cells (**Figure 3**). The patient continued treatment with brigatinib, and there has been no evidence of recurrence until the last follow-up, 13 months post-surgery. Additionally, the patient did not experience any brigatinib-related adverse events, and there were no notable findings in either the tumor marker or biochemistry laboratory test results. **Figure 4** illustrates the timeline of the entire therapy process.

## 4 Discussion

To the best of our knowledge, the current case report represents the first instance of a patient achieving a pCR after neoadjuvant brigatinib therapy for stage III ALK-positive NSCLC. This not only highlights the safety and feasibility of subsequent surgical intervention but also provides robust pathological evidence of the potent antitumor efficacy of brigatinib.

ALK-TKIs have markedly improved outcomes in patients with advanced ALK-positive NSCLC, who account for 2–7% of all NSCLC cases (4, 12). Traditionally, the treatment strategy for early-stage ALK-positive NSCLC has been similar to that for NSCLC without oncogenic driver mutations, achieving a 5-year survival rate for stage IIIA NSCLC of only 36% (1). However, promising results from recent applications of advanced NSCLC therapies in early-stage, perioperative settings are shifting this paradigm (13). Moreover, historical studies have demonstrated encouraging responses to ALK-TKIs in the neoadjuvant setting, leading to ongoing research, such as the ALNEO and NAUTICA1 trials (7–9, 14, 15). The results of these studies are promising and suggest potential changes in treatment strategies that could offer more personalized options for these patients. In Korea, reimbursement is provided for the use of first-line ALK-TKIs, and neoadjuvant ALK-TKI treatment has been selected through multidisciplinary consultation. Consequently, the implementation of comprehensive molecular pathology testing could further enhance treatment efficacy in early-stage NSCLC (16).

A dramatic radiologic response to brigatinib therapy has been commonly observed in patients with advanced ALK-positive NSCLC ( (10, 17). Based on historical analyses, brigatinib is predicted to exert the most expansive range of efficacy against *ALK* mutations, with encompassing activity against secondary mutants predicted to confer high resistance to ceritinib or alectinib (18–20). Recently, Zhang et al. reported the efficacy of alectinib in patients with N2 NSCLC, with 11 patients (91%) showing a major pathological response. Moreover, two patients (18.2%) achieved a pCR (8). Furthermore, Zenke et al. reported on the efficacy of ceritinib in patients with stage IIIA NSCLC, with four patients (57%) showing a major pathological response and two patients (28%) achieving a pCR (9). However, no previous case report has documented pathological evidence of a pCR achieved with brigatinib therapy. The current case report suggests that neoadjuvant brigatinib is both effective and safe in resectable stage III ALK-positive NSCLC.

Multiple questions regarding the use of ALK-TKIs in early-stage NSCLC need to be addressed. The duration of neoadjuvant therapy and the optimal timing of surgery remain unclear. Clinical trials are needed to assess the safety and efficacy of neoadjuvant therapy. Additionally, it needs to be established whether maintenance therapy with ALK-TKIs is effective for patients with ALK-positive NSCLC who have shown favorable responses in the neoadjuvant setting.

## 5 Conclusion

In conclusion, we presented a case of ALK-positive NSCLC with a pCR in resected specimens after treatment with brigatinib. Conversion surgery after brigatinib therapy may be safe and effective in patients with ALK-positive NSCLC.

## Data availability statement

The original contributions presented in the study are included in the article materials. Further inquiries can be directed to the corresponding author.

## Ethics statement

The studies involving humans were approved by Institutional Review Board of Pusan National University Hospital (IRB no. 2310-017-131). The studies were conducted in accordance with the local legislation and institutional requirements. The participants provided their written informed consent to participate in this study. Written informed consent was obtained from the individual(s) for the publication of any potentially identifiable images or data included in this article.

## Author contributions

HS: Writing – review & editing, Writing – original draft, Methodology, Conceptualization. SK: Writing – original draft, Validation, Data curation. MK: Writing – original draft, Supervision, Investigation. JC: Writing – review & editing, Supervision, Resources. AK: Writing – review & editing, Supervision, Resources. JE: Writing – review & editing.
